# Using Machine Learning to Analyze the Predictors of Life Satisfaction: Focus on Lifestyle Attitudes and Psychological Factors

**DOI:** 10.1002/mpr.70051

**Published:** 2026-04-01

**Authors:** Furkan Bahadir Alptekin, Ebrar Torlak, Özge Asik, Betul Karaaslan, Ebru Turgal, Huseyin Sehit Burhan, Hasan Mervan Aytac, Oya Guclu

**Affiliations:** ^1^ Department of Psychiatry Basaksehir Cam and Sakura City Hospital University of Health Sciences Istanbul Turkey; ^2^ Faculty of Medicine, Department of Biostatistics Ankara University Ankara Turkey; ^3^ Institute of Graduate Studies in Health Sciences Istanbul University Istanbul Turkey

## Abstract

**Objectives:**

Life satisfaction is an essential indicator of quality of life, and enhancing it can contribute to individual well‐being strategies. Because it is a complex concept, a comprehensive approach is needed to address it effectively. Machine learning offers a unique statistical opportunity to address this challenge effectively. In this study, we examined how lifestyle parameters, psychological issues, and psychological processes predict life satisfaction.

**Methods:**

The study included 1366 participants, representing the general population. Lifestyle factors were self‐reported, and included exercise frequency, alcohol consumption, smoking, body mass index, and regularity of social rhythms. The participants also completed several assessment scales, such as the Life Satisfaction Scale, the Hospital Anxiety and Depression Scale, the Acceptance and Action Questionnaire–II, the Tuckman Procrastination Scale, the Big Three Perfectionism Scale–Short Form, and the Brief Social Rhythm Scale. Machine‐learning methods were used to evaluate the statistical parameters, with root mean square error values of 3.9, 3.6, and 3.7 for gradient boosting, extreme gradient boosting, and light gradient‐boosting machine, respectively.

**Results:**

The top five factors influencing life satisfaction were identified as depression scores, psychological inflexibility, marital status, social rhythm, and procrastination. Psychological inflexibility influences the impact of depression on life satisfaction. Factors that are difficult or impossible to change, such as age, gender, education, and chronic disease, ranked lower on the list. By contrast, psychological and environmental factors that can be improved had strong predictive power.

**Conclusions:**

These findings offer opportunities for enhancing life satisfaction and underscore the responsibility to address these factors.

## 1 Introduction

One of the most fundamental questions individuals ask about their lives is whether they are satisfied with them. The majority of their efforts are aimed at improving their life satisfaction. Life satisfaction, as described by Miller, is a cognitive evaluation process in which individuals subjectively assess the quality of their lives based on unique criteria (Miller 2011). It is widely considered the most important indicator of well‐being (Charlemagne‐Badal et al. 2015). Life satisfaction is a complex phenomenon and is related to several factors. Many interconnected or unconnected factors have been shown to be related to life satisfaction. These include sociodemographic aspects, such as age, gender, psychological features, lifestyle, and involvement in leisure activities (Tokay Argan and Mersin 2021). The complex and multifaceted nature of the factors influencing life satisfaction has led researchers to categorize them under specific headings. Branc‐Allen et al. classified these factors into three groups: exogenous factors (such as age, gender, and marital status), intervening factors (like social interactions with friends or participation in leisure activities), and endogenous factors (which relate to internal processes) (Branch‐Allen and Jayachandran 2016). Similarly, developed a comparable classification using different terminology. In this framework, personality traits align with internal processes, life circumstances are considered exogenous factors, and intentional activities are categorized as intervening factors (Lyubomirsky et al. 2005). Lifestyle attitudes and healthy behaviors, as intentional activities, have emerged as an important research area in recent years, with demonstrated effects on life satisfaction across different age groups (Durand‐Sanchez et al. 2023; Grant et al. 2009; Pengpid and Peltzer 2019). Endogenous concepts and personality traits—including psychological inflexibility, procrastination, perfectionism, and an individual's mood—can also impact lifestyle attitudes. Lifestyle is all behaviors that are under the control of individuals and affect their health risks (Avsar and Kizilaslan 2025). It is apparent that lifestyle attitudes are one of the parameters that impact life satisfaction, with several studies having established a connection between life satisfaction and lifestyle attitudes, such as eating habits, physical activity, smoking, alcohol consumption, and body mass index (BMI, calculated as weight in kilograms divided by height in meters squared) (Ayhan et al. 2021; Phulkerd et al. 2021; Velten et al. 2014). However, the strength of the correlations is variable in these studies. The results have led to the examination of psychological processes and problems that may be related to lifestyle attitudes and have shown a contribution to life satisfaction. Among these, depression, anxiety, psychological inflexibility, procrastination, and perfectionism are at the fore.

Numerous psychological problems, such as anxiety and depressive disorders, have been identified as having a negative impact on life satisfaction (Mamani‐Benito et al. 2022; Meule and Voderholzer 2020; Ooi et al. 2022). Also, growing evidence shows that lifestyle attitudes are related to depression. There has been a considerable amount of study on both the mutual effects of lifestyle attitudes and depression and lifestyle changes as a treatment for depression (Lopresti et al. 2013; Raboch et al. 2017; Sánchez‐Villegas et al. 2016; Sarris et al. 2014).

A model that explains the development of psychopathology is the psychological flexibility/psychological inflexibility (PI) model. Psychological flexibility is defined as “the ability to fully contact the present moment and the thoughts and feelings it contains without needless defense… [and] persisting or changing behavior in pursuing goals and values” (Lucas and Moore 2020). Conversely, PI is a pattern where behavior is overly controlled by one's thoughts, emotions, and other internal experiences, or these experiences are avoided at the expense of more effective and meaningful actions (Levin et al. 2014; Lucas and Moore 2020). Both in the general population and specific groups, PI has been linked to psychiatric disorders and life satisfaction, and is a transdiagnostic process related to lifestyle attitudes (Cyniak‐Cieciura et al. 2023; Lucas and Moore 2020; Wicksell et al. 2008). Based on a systematic review, Acceptance and Commitment Therapy (ACT), which aims to increase psychological flexibility, helps maintain long‐term lifestyle and behavioral changes, such as weight management, effective coping with substance‐related and addictive problems, eating, and physical activity (Yıldız 2020).

Procrastination is characterized by the voluntary postponement of a planned course of action, even though the individual anticipates being disadvantaged due to the delay (Johansson et al. 2023). It is one of the essential processes related to different aspects of lifestyle attitudes (Hammoudi et al. 2021; Kelly and Walton 2021). Higher levels of procrastination are associated with lower life satisfaction. One study that examined the relationship between procrastination, physical activity, and quality of life through a holistic perspective found that regular physical activity correlates with a more positive perception of quality of life, which in turn is associated with lower levels of procrastination (Codina et al. 2020).

Perfectionism typically reflects an individual's drive to achieve perfection or maintain the illusion of perfection, which involves maintaining unattainable standards of excellence and expecting flawless performance (Holden and Jeanfreau 2023). Because of its composite characteristics, perfectionism has complex associations with life satisfaction and lifestyle attitudes. Dissatisfaction might be viewed as a hallmark of high perfectionism because perfectionists aspire to achieve perfection in every aspect of their lives, resulting in ongoing dissatisfaction across various life domains. However, previous studies have found that life satisfaction is positively and negatively correlated with different dimensions of perfectionism (Liu et al. 2022). There has been a limited number of studies and complicated results concerning the relationships between perfectionism and eating behaviors, exercise performance, and substance use (i.e., smoking and drinking) (Damián et al. 2024; Nelsen et al. 2023; Pratt et al. 2023).

As is currently understood, life satisfaction is a complex concept, with interwoven variables impacting it non‐linearly. Due to diminishing returns, improvement in less satisfying variables could yield more significant overall life satisfaction gains than in already high‐satisfaction domains. Therefore, efforts to enhance life satisfaction should consider all variables, prioritizing those with lower satisfaction levels rather than focusing solely on highly valued domains. This situation calls for a comprehensive approach in the research area, emphasizing overall life satisfaction rather than isolated domain satisfaction (Rojas 2007).

The complex nature of life satisfaction, which requires a comprehensive approach, presents a challenge in forming a hypothesis to examine the related factors. Machine learning (ML) offers a unique statistical opportunity to effectively address this challenge. Such methods, when used in clinical psychology and psychiatry, are specifically designed to learn statistical functions from multidimensional datasets. Machine learning allows for the generation of generalizable predictions about individuals. Traditional statistics that focus on explanations are inadequate for research designs with a multifactorial structure (Dwyer et al. 2018), whereas ML statistics have a distinct advantage in their ability to handle the complexity of psychological data (Coutanche and Hallion 2020).

This study draws on the conceptual frameworks of Branc‐Allen et al. and Lyubomirsky et al. to examine how various lifestyle factors—such as exercise, smoking, alcohol consumption, body mass index, and daily rhythms—affect life satisfaction. Additionally, we aim to investigate the predictive influence of lifestyle attitudes and related psychological factors, including anxiety, depression, psychological inflexibility, procrastination, and perfectionism, along with several external factors, on life satisfaction.

## 2 Methods

### 2.1 Participants and Procedure

We recruited participants through invitations distributed on social media platforms, and their data were collected via Google Forms. The inclusion criteria were individuals aged between 18 and 65 years with no mental or physical illnesses that hindered their ability to complete the questionnaire. Prior to taking part, the participants provided their informed consent. A total of 1653 people were contacted. However, five individuals declined to participate and 282 were not included because their answers were incomplete. Finally, 1366 participants, who had both consented and completed the questionnaire, were included. The study adhered to the principles outlined in the Research Helsinki Declaration, and necessary approval was granted by the Başakşehir Çam and Sakura City Hospital Clinical Research Ethics Committee (2023‐400).

### 2.2 Instruments

*The Sociodemographic Information Form* includes participants' educational and professional status, information about chronic diseases, height, and weight. Lifestyle attitudes, smoking, alcohol use status, and exercise frequency were assessed using a five‐point Likert‐type scale, similar to that used by (Velten et al. 2014).

*The Life Satisfaction Scale (LSS)* developed by consists of five questions answered through a five‐point Likert scale (Diener et al. 1985). Higher scores indicated greater life satisfaction. The Turkish validity and reliability were established by Dağlı and Baysal in 2016 (Dağli and Baysal 2016). In this study, the Cronbach's alpha coefficient for this scale was 0.86. Composite reliability (CR) = 0.86, and average variance extracted (AVE) = 0.55, supporting convergent validity. Model fit indices demonstrated good fit: Comparative Fit Index (CFI) = 0.99, Tucker–Lewis Index (TLI) = 0.99, Root Mean Square Error of Approximation (RMSEA) = 0.05, Standardized Root Mean Square Residual (SRMR) = 0.01.

*The Brief Social Rhythm Scale*, developed by Margraf et al., consists of 10 items assessing perceived social rhythm related to sleep, eating, waking time, and social relations (Margraf et al. 2016). Each item was answered on a six‐point Likert scale. Higher scores indicated greater irregularity in social rhythm. The measure showed ggod or acceptable psychometric properties (*α* = 0.84, CR = 0.82, AVE = 0.35). However, confirmatory factor analysis showed poor fit indices: CFI = 0.49, TLI = 0.35, RMSEA = 0.30, SRMR = 0.20.

*The Hospital Anxiety and Depression Scale* was designed to measure anxiety and depression in a general medical population (Zigmond and Snaith 1983). It consists of 14 self‐completed items––seven for anxiety and seven for depression––with high scores suggesting increased levels of both. Aydemir et al. conducted a validity and reliability study in 1997 (Aydemir et al. 1997). The depression subscale showed acceptable reliability (*α* = 0.78, CR = 0.78) and convergent validity (AVE = 0.35), with optimal model fit (CFI = 0.98, TLI = 0.97, RMSEA = 0.05, SRMR = 0.03). The anxiety subscale similarly exhibited good reliability (*α* = 0.85, CR = 0.86) and validity (AVE = 0.48), with good model fit indices (CFI = 0.97, TLI = 0.96, RMSEA = 0.07, SRMR = 0.03). These results support the use of both HADS subscales in the current sample.

*The Acceptance and Action Questionnaire–II* measures PI (Bond et al. 2011). It consists of seven items rated on a seven‐point Likert scale that evaluate experiential avoidance, with higher scores indicating greater PI. Yavuz et al. adapted it for Turkey in 2016 (Yavuz et al. 2016). The scale demonstrated strong psychometric properties with *α* = 0.85, CR = 0.90, and AVE = 0.57, indicating adequate reliability and convergent validity. The model fit indices showed acceptable initial fit. Based on modification indices and theoretical considerations, error covariances were added between items with similar wording (Items 1 and 1, Item 2 and 3, and Items 6 and 7) The revised model demonstrated excellent fit (Before revision: CFI = 0.91, TLI = 0.87, RMSEA = 0.16, SRMR = 0.05; after revision: CFI = 0.99, TLI = 0.98, RMSEA = 0.04, SRMR = 0.02).

*The Tuckman Procrastination Scale,* originally comprising 16 items rated on a four‐point scale (Tuckman 1991), was adapted for Turkish use, resulting in 14 items and a five‐point Likert scale (Özer et al. 2013). Higher scores indicate increased procrastination. Internal consistency was good (*α* = 0.81), and construct validity was supported by CR = 0.88 and AVE = 0.50. The CFA model exhibited acceptable fit indices (CFI = 0.93, TLI = 0.91, RMSEA = 0.09, SRMR = 0.04).

The *Big Three Perfectionism Scale–Short Form*, developed by Feher et al., comprises 16 items with a three‐factor model. It assesses rigid perfectionism, self‐critical perfectionism, and narcissistic perfectionism, with total scores ranging from 16 to 80 (Feher et al. 2020). Kaçar‐Başaran et al. conducted the Turkish adaptation (Kaçar‐Başaran et al. 2022). Reliability analysis yielded *α* = 0.89, with CR = 0.90 and AVE = 0.38 exceeding recommended thresholds. Model fit statistics indicated acceptable of poor fit to the data: CFI = 0.83, TLI = 0.80, RMSEA = 0.10, SRMR = 0.07. The scale is used with total scores in the study.

### 2.3 Statistical Analysis

The sociodemographic information was evaluated from the percentage, mean, and standard deviation values. Pearson's correlation analysis was performed to determine the relationship between life satisfaction and the dependent variables.

The ML analyses were conducted using the h2o R package, extreme gradient boosting (XGBoost), and light gradient‐boosting machine (LightGBM).

During the analysis, the predictors were systematically assigned categorical, ordinal, or continuous status, as dictated by their inherent characteristics and by the appropriate statistical methodologies applied. Table 1 contains the descriptions of the dataset variables. Recent work emphasizes that tree‐ensemble regression models typically need far larger samples than simple “10‐per‐predictor” rules suggest, especially in psychology/behavioral data. Empirical and simulation studies indicate that stable GBM/XGBoost/LightGBM performance often requires on the order of 100–1000 cases. For example, one simulation found that even under ideal conditions a GBM only reached maximal predictive accuracy at roughly *N* ≈ 1000 (Damar et al. 2025). Likewise, Zantvoort et al. (2024) observed that *N* = 500 “mitigated overfitting” but performance did not converge until ≈750–1500, leading to a practical recommendation of ≈*N* = 1000 as a minimum dataset size (Zantvoort et al. 2024).

**TABLE 1:** Machine‐learning model variable descriptions.

Variable	Description	Value
Life satisfaction	Score of LSS	1–25, higher scores indicate greater life satisfaction
Depression	Score of HADS/Depression part	0–42, higher scores indicate greater depression
Anxiety	Score of HADS/Anxiety part	0–42, higher scores indicate greater anxiety
PI	Psychological inflexibility/Score of AAQ‐II	1–49, higher scores indicate greater psychological inflexibility
Procrastination	Score of TPS	14–80, higher scores indicate greater procrastination
Social rhythm	Score of BSRS	0–60, higher scores indicate greater irregularity in social rhythm
Perfectionism	Score of BTPS‐16	16–80, higher scores indicate greater perfectionism
Marriage status		0: Married, 1: Single
Age		Coninuous variable
Sex		1: Male, 2: Female
BMI	kg/m^2^	Coninuous variable
Exercise frequency		A:1, b:2, c:3, d:4, e:5
Education	Educational status	Primary school:1, Secondary school:2, High school:3, Graduate:4, Masterate or doctorate:5
Alcohol	Alcohol consumption frequency	No:1, 1 time per month:2, 2–4 times a month:3, 2–3 times a week:4, 4 or more times a week:5
Smoking	Smoking frequency	No:1, Sometimes:2, less than half a pack a day:3, half a day—1 pack:4, more than 1 pack per day:5
Chronic disease		Yes:1, No:2

Abbreviations: AAQ‐II, Acceptance and Action Questionnaire‐II; BMI, body mass index; BSRS, Brief Social Rhythm Scale; BTPS‐16, Big Three Perfectionism Scale—Short Form; HADS, Hospital Anxiety and Depression Scale; LSS, Life Satisfaction Scale; PI, Psychological Inflexibility; TPS, Tuckman Procrastination Scale.

#### 2.3.1 Machine‐Learning Methods

Three ML algorithms underwent refinement to enhance the model predictions for life satisfaction. All three models considered a maximum set of three decision trees. A shrinkage rate of 0.3 was also selected for the tuning process. Using the createDataPartition function in R, 80% of the dataset was selected as the training data and was separated out. This portion was assigned to the variable training data. The remaining 20% of the dataset was further divided into two parts. The first part was designated the validation data and was assigned to be the variable validation data. The remaining part was separated out and assigned to be the variable test data. This approach created a three‐way split in the dataset––training, validation, and testing. Such a division is commonly used in ML to train, validate, and finally test models. This division allows for an evaluation of the model's performance on both the training and validation datasets before its generalization on the test dataset is assessed. The performance of each model was assessed using the mean absolute error (MAE) and the root mean square error (RMSE), the metric results being displayed in Table 2.

**TABLE 2:** Test metrics using ensemble approaches.

Set	Metric	Gradient boosting	Extreme gradient boosting (XGBoost)	LightGBM
Test	MAE	28,997,469	2.825757	2.864427
	RMSE	36,491,566	3.657236	3.733067
	*R* ^2^	0.270	0.268	0.243
Training	MAE	2,150,779	2493	2211
	RMSE	274,172	3166	2829
	*R* ^2^	0,621,273	0.495	0.593

Abbreviations: MAE, mean absolute error; RMSE, root‐mean‐square error.

Tree‐ensemble methods like gradient boosting (including XGBoost and LightGBM) have proven highly effective for tabular behavioral data, often exceeding the accuracy of linear or kernel methods. For example, in large social‐survey studies of well‐being (with on the order of 10 predictors) gradient boosting consistently achieved the highest out‐of‐sample *R* ^2^, outperforming both ordinary least squares and random forests (Oparina et al. 2025). These boosted trees naturally accommodate mixed predictor types LightGBM even handles categoricals natively to reduce overfitting (Ooba et al. 2023). In practice this means one can achieve “black‐box”–level accuracy without losing transparency—for instance, an XGBoost + SHAP analysis on personality and demographic data both maximized predictive AUC and revealed the direction and magnitude of each trait's effect on honest behavior (Meng et al. 2025). By contrast, alternative algorithms have notable drawbacks in this setting. Random forests are also robust and handle mixed data, but tend to yield slightly lower accuracy than optimized boosting. kNearest Neighbors is simple but struggles with irrelevant dimensions and offers no global model interpretation. Neural networks (even small feedforward nets) generally demand larger samples and custom tuning; in fact, one recent analysis found that a basic neural net showed no predictive gain over linear regression on psychological survey outcomes (Oparina et al. 2025). In sum, for regression tasks with on the order of 10–20 predictors, gradient‐boosted trees (XGBoost/LightGBM) strike an excellent balance: they achieve top predictive accuracy with typical behavioral‐science sample sizes, natively handle heterogenous and noisy inputs, and remain interpretable through post‐hoc methods like SHAP.

#### 2.3.2 Feature Selection

The reason we used different ML methods was that they can give varying performances depending on various factors, such as feature dimension, data separability, data balancing, and feature correlation. To select the important features affecting life satisfaction, we investigated the contribution of each of the 15 input variables on severity through feature importance analysis using the XGBoost, gradient‐boosting machine (GBM), and LightGBM algorithms. For hyperparameters, the best combination was determined by performing a grid search for “max_depth,” “eta,” “num_leaves,” and “nrounds.” The explanations for these abbreviations are as follows:

max_depth: This parameter specifies the maximum depth of the trees in the model. A higher value allows the model to capture more complex patterns, but can also lead to overfitting.

eta (also known as learning_rate): This parameter controls the step size at each iteration while moving toward the optimal solution. Lower values make the model learn more slowly, which can improve accuracy, but requires more iterations.

nrounds (also known as ntrees): This parameter represents the number of boosting iterations or trees built in the model. More rounds generally lead to better performance, but can increase the computational cost.

num_leaves: This parameter specifies the number of leaves in each tree. Increasing the number of leaves can make the model more complex and more capable of capturing intricate patterns.

The model was then retrained using the best hyperparameters. This process was performed separately for each ML algorithm. For XGBoost, we set the hyperparameters as follows: max_depth to 3, 5, and 7; eta (learning_rate) to 0.1, 0.01, and 0.001; and nrounds to 25, 50, and 100. For LightGBM, we set the hyperparameters as follows: max_depth to 3, 5, and 7; learning_rate to 0.1, 0.01, and 0.001; and num_leaves to 25, 50, and 100. For the GBM in h2o, we set the hyperparameters as follows: ntrees to 25, 50, and 100; max_depth to 3, 5, and 7; and learning_rate to 0.1, 0.01, and 0.001.

**FIGURE 1:**
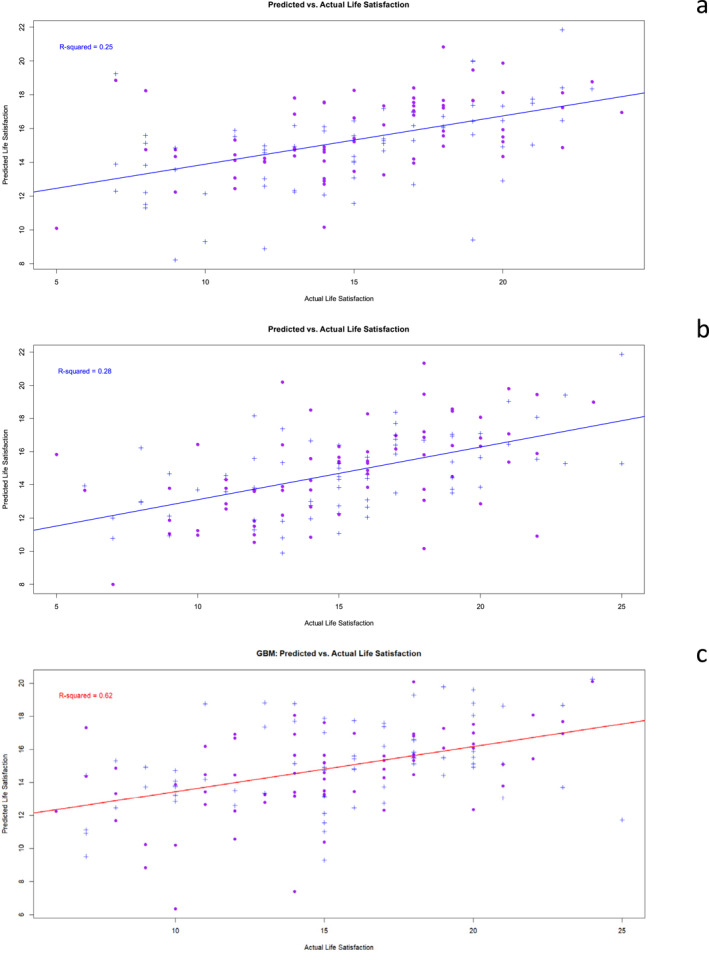
Model performance: Predicted versus actual values of: (a) lightGBM; (b) XGBoost; and (c) GBM.

In addition, the actual versus predicted life satisfaction values were evaluated using the coefficient of determination (*R* ^2^) and are presented graphically (for lightGBM, *R* ^2^ = 0.25; for XGBoost, *R* ^2^ = 0.28; for GBM, *R* ^2^ = 0.62) (see Figure 1).

We showed which parameters contributed to determining the life satisfaction scores using SHapley Additive exPlanations (SHAP) values, which indicate the contribution of each feature to the model result. An expected output value from the model for the trained dataset exists, known as the base value. The contribution of the SHAP values to the model shows how far the model has moved away from this base value. Features that contribute more are considered features that are important to the model. These contributions can be negative or positive (Stadtler et al. 2022).

#### 2.3.3 Explainability Analysis

To examine whether the machine‐learning models captured non‐linear and interactive effects among predictors, SHAP values were computed for each model using the shapviz package in R. SHAP dependence and interaction plots were generated to visualize how changes in one predictor influenced the predicted life satisfaction score, conditional on another variable. This approach quantifies both main effects and higher‐order interactions in a model‐agnostic, interpretable manner.

## 3 Results

### 3.1 Sample Characteristics

Of the 1366 participants, 80.4% (*n* = 1098) were women. The average age was 33.8 years. Forty‐nine percent were married, while the rest were either single or divorced. Over 75% of the participants had a university education or higher. Table 3 presents the descriptive statistics of the variables analyzed for the sample.

**TABLE 3:** Sociodemographic variables.

		*n*	**%**	*M*	SD
Age				33.78	11.457
Sex					
	Male	268	80.38		
	Female	1098	19.62		
Marital status					
	Married	669	48.98		
	Single	697	51.02		
Education					
	Primary school	58	4.24		
	Secondary school	403	29.50		
	High school	562	41.14		
	Graduate	275	20.13		
	Masterate or doktorate	68	4.98		
Smoking					
	No	897	65.66		
	Sometimes	163	11.93		
	Less than half a pack a day	99	7.24		
	Half a day—1 pack	167	12.22		
	More than 1 pack per day	40	2.92		
Alcohol consumption					
	No	835	61.12		
	1 time per month	234	17.13		
	2–4 times a month	215	15.73		
	2–3 times a week	63	4.61		
	4 or more times a week	19	1.39		
Chronic disease					
	Yes	249	18.22		
	No	1117	81.77		

Abbreviations: M, mean; SD, standard deviation.

### 3.2 Correlations

Table 4 displays the bivariate correlations among the variables. Life satisfaction showed a significant relationship with all parameters, except for alcohol use. Importantly, it demonstrated a moderate correlation with depression (*r* = −0.480, *p* < 0.01), anxiety (*r* = −0.409, *p* < 0.01), PI (*r* = −0.438, *p* < 0.01), procrastination (*r* = −0.317, *p* < 0.01), and social rhythm (*r* = −0.364, *p* < 0.01). There was a small correlation with age (*r* = 0.230, *p* < 0.01), perfectionism (*r* = −0.215, *p* < 0.01), and exercise frequency (*r* = 0.138, *p* < 0.01). Depression showed a strong correlation with anxiety (*r* = 0.606, *p* < 0.01) and PI (*r* = 0.570, *p* < 0.01). Additionally, a moderate correlation with depression was observed between procrastination (*r* = 0.336, *p* < 0.01), perfectionism (*r* = 0.335, *p* < 0.01), and social rhythm (*r* = 0.473, *p* < 0.01).

**TABLE 4:** Bivariate correlation of continuous variables.

	*M*	SD	1	2	3	4	5	6	7	8	9	10	11
1. Life satisfaction	15.09	4.43	—										
2. Depression	6.91	4.00	−0.480^d^	—									
3. Anxiety	8.76	4.34	−0.409^d^	0.606^d^	—								
4. PI	24.66	10.74	−0.438^d^	0.570^d^	0.661^d^	—							
5. Procrastination	37.88	11.80	−0.317^d^	0.336^d^	0.352^d^	0.389^d^	—						
6. Perfectionism	47.97	48.00	−0.215^d^	0.335^d^	0.479^d^	0.471^d^	0.246^d^	—					
7. Social rhythm^a^	29.86	29.00	−0.364^d^	0.473^d^	0.419^d^	0.411^d^	0.376^d^	0.285^d^	—				
8. Exercise frequency^b^	1.71	1.00	0.138^d^	−0.164^d^	−0.160^d^	−0.138^d^	−0.170^d^	−0.143^d^	−0.188^d^	—			
9. Alcohol^b^	1.68	1.00	−0.001	−0.037	0.059^d^	0.024	0.090^a^	0.048	0.030	0.067^c^	—		
10. Smoking^b^	1.75	1.00	−0.05	0.072^d^	0.102^a^	0.101^d^	0.014	0.025	0.107^d^	−0.043	0.366^d^	—	
11. BMI	23.98	4.55	0.06^c^	0.060^c^	−0.063^c^	−0.018	−0.012	−0.048	−0.037	−0.006	−0.160^d^	−0.065^c^	—
12. Age	33.78	11.45	0.23^d^	−0.166^d^	−0.257^d^	−0.221^d^	−0.295^d^	−0.215^d^	−0.186^d^	−0.097^d^	−0.059^c^	−0.091^d^	0.364^d^

*Note:* Assessed with Pearson correlation.

Abbreviations: BMI, body mass index; M, mean; PI, psychological inflexibility; SD, standard deviation.

^a^ increased scores indicate irregular social life.

^b^ measured with a 5‐point Likert type question.

^c^ Correlation is significant at the 0.05 level (2‐tailed).

^d^ Correlation is significant at the 0.01 level (2‐tailed).

### 3.3 Discovery Sample Model Performance

The metric MAE and RMSE results are displayed in Table 2. A small value of RMSE might be interpreted as a better model (Salkind 2010). In addition, the actual versus predicted life satisfaction values were evaluated using *R* ^2^ and are presented graphically (for lightGBM, *R* ^2^ = 0.25; for XGBoost, *R* ^2^ = 0.28; and for GBM, *R* ^2^ = 0.62) (Figure 1). Based on these results, the ML algorithms were performing well.

### 3.4 Variable Importance

**FIGURE 2:**
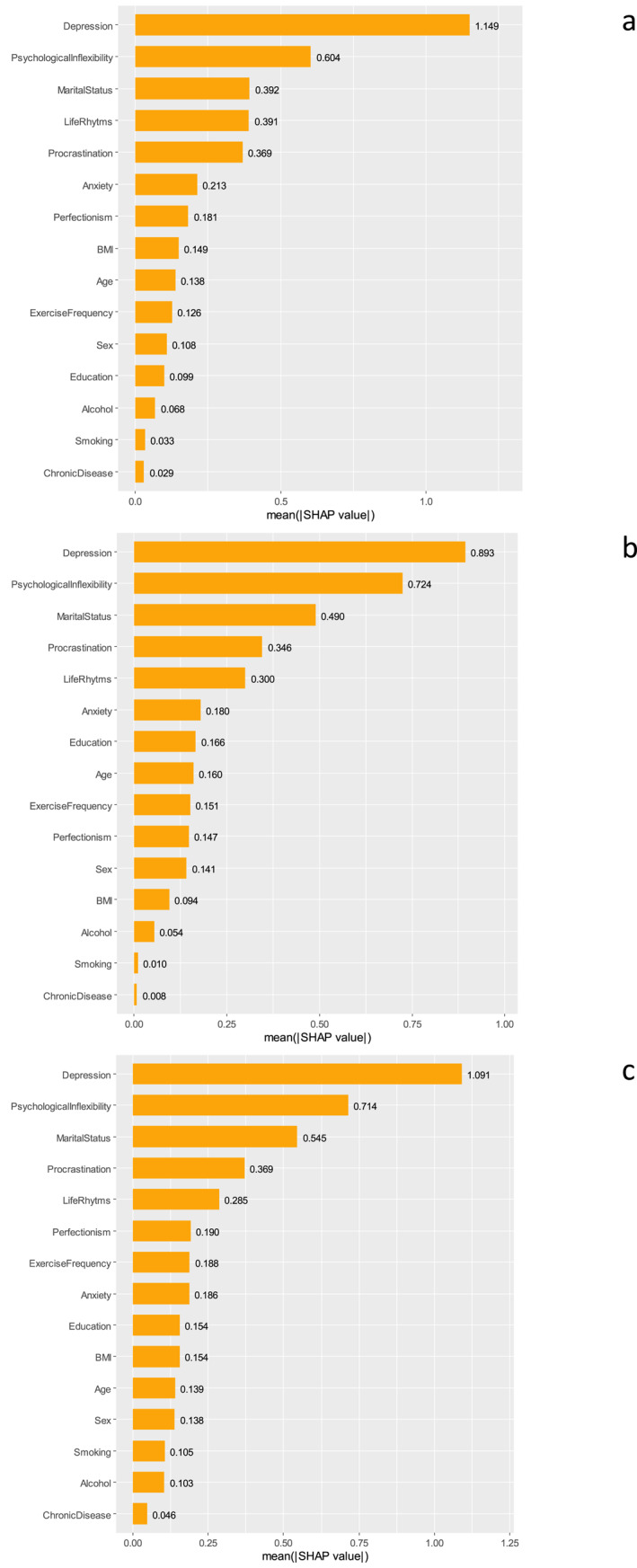
Feature importance bar plot of life satisfaction estimated by the SHapley Additive exPlanation (SHAP) method on: (a) LightGBM; (b) XGBoost; and (c) GBM.

**FIGURE 3:**
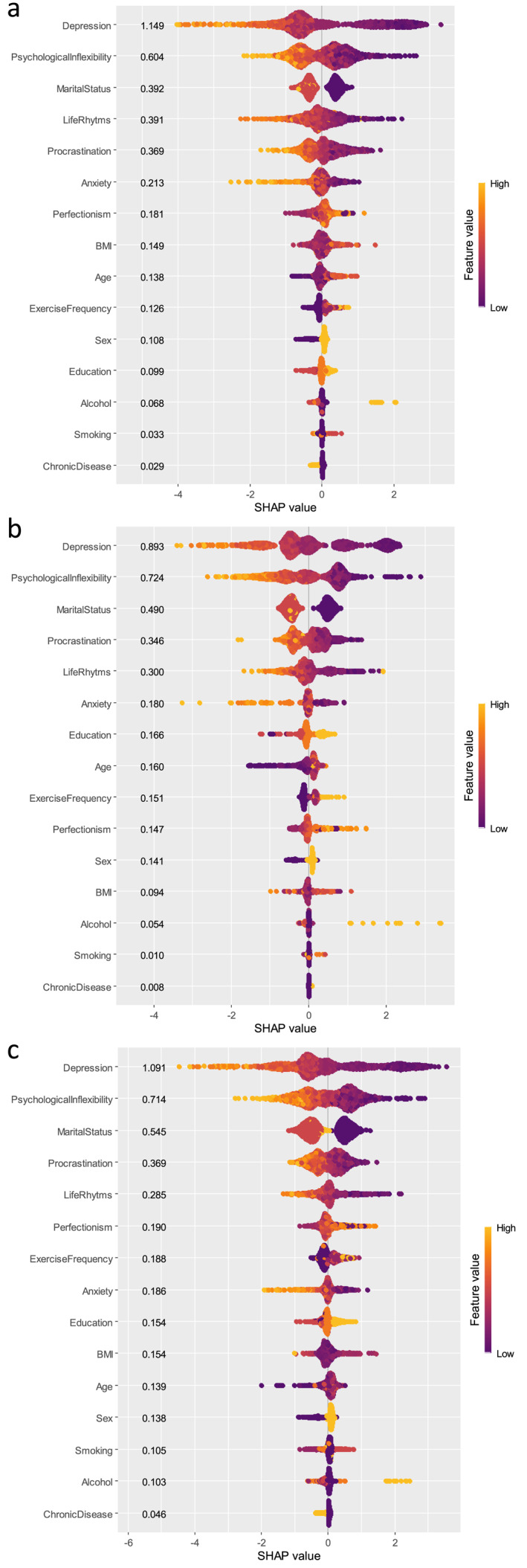
SHAP summary plots of life satisfaction from: (a) LightGBM; (b) XGBoost; and (c) GBM. Cases with high values are shown in red, those with low values in blue. The variables are ranked in descending order.

We evaluated all the predictive variables for life satisfaction using the 3 ML algorithms (GBM, XGBoost, LightGBM) to examine and comprehend their respective levels of impact. Figure 2 presents the mean SHAP values, representing the average effect of each variable on the model for the 3 ML methods. Additionally, we present a SHAP summary plot for all 3 ML methods (Figure 3). Each feature's contribution to the model for each participant is represented by a dot, with each participant assigned a dot for each feature. The dots are colored based on the participant's corresponding feature values, accumulating vertically to indicate density. Yellow represents higher feature values, while purple represents lower feature values. The three most predictive variables for the three algorithms were depression, psychological inflexibility, and marital status. Although their places in the rankings changed, the fourth and fifth factors were the same for all three models––procrastination and life rhythm. Age, sex, perfectionism, exercise frequency, anxiety, education, BMI, chronic diseases, smoking, and alcohol consumption were the other variables with different, but weaker, predictive effects in the different algorithms.

**FIGURE 4:**
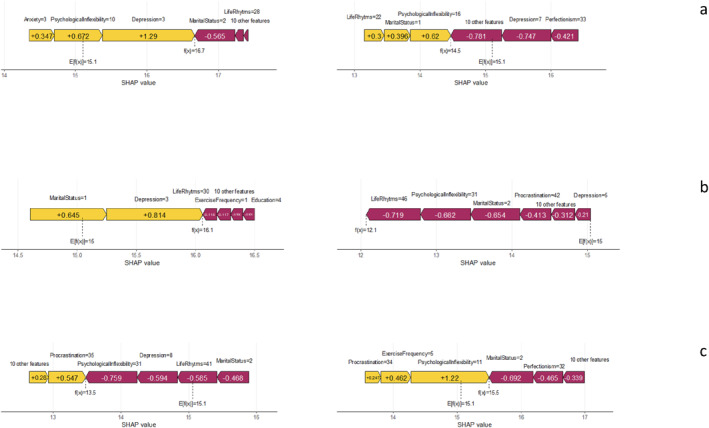
Explanation of the first and sixteenth individuals using SHAP force graphs via: (a) LightGBM; (b) XGBoost; and (c) GBM.

We formed the force plot from the first and sixteenth individuals of all the algorithms to view the local interpretability (selected randomly), explaining the predictions for individual data instances. This is a highly intuitive approach that produces simple, but informative, outputs. In Figure 4a, the LightGBM model's predicted value is 15.1 and the logit transformation for the first individual without modification is 16.7. It can also be seen from Figure 4a that the model's predicted value is 15.1 and the logit transformation for the 16th individual without any modification is 14.5. Additionally, the yellow color shows that the feature increases life satisfaction, while the purple color shows that the feature decreases life satisfaction. Features in the yellow region indicate that these push life satisfaction to higher values. The force plot from XGBoost and GBM are shown in Figure 4b and 4c, respectively.

### 3.5 Feature Interpretation

The SHAP interaction plots on LightGBM, XGBoost, and Gradient Boosting revealed that depression exerted a strong negative non‐linear impact on predicted life satisfaction, with SHAP values decreasing sharply as depression scores increased, and the negative effect steepening particularly at moderate‐to‐high depression levels.

**FIGURE 5:**
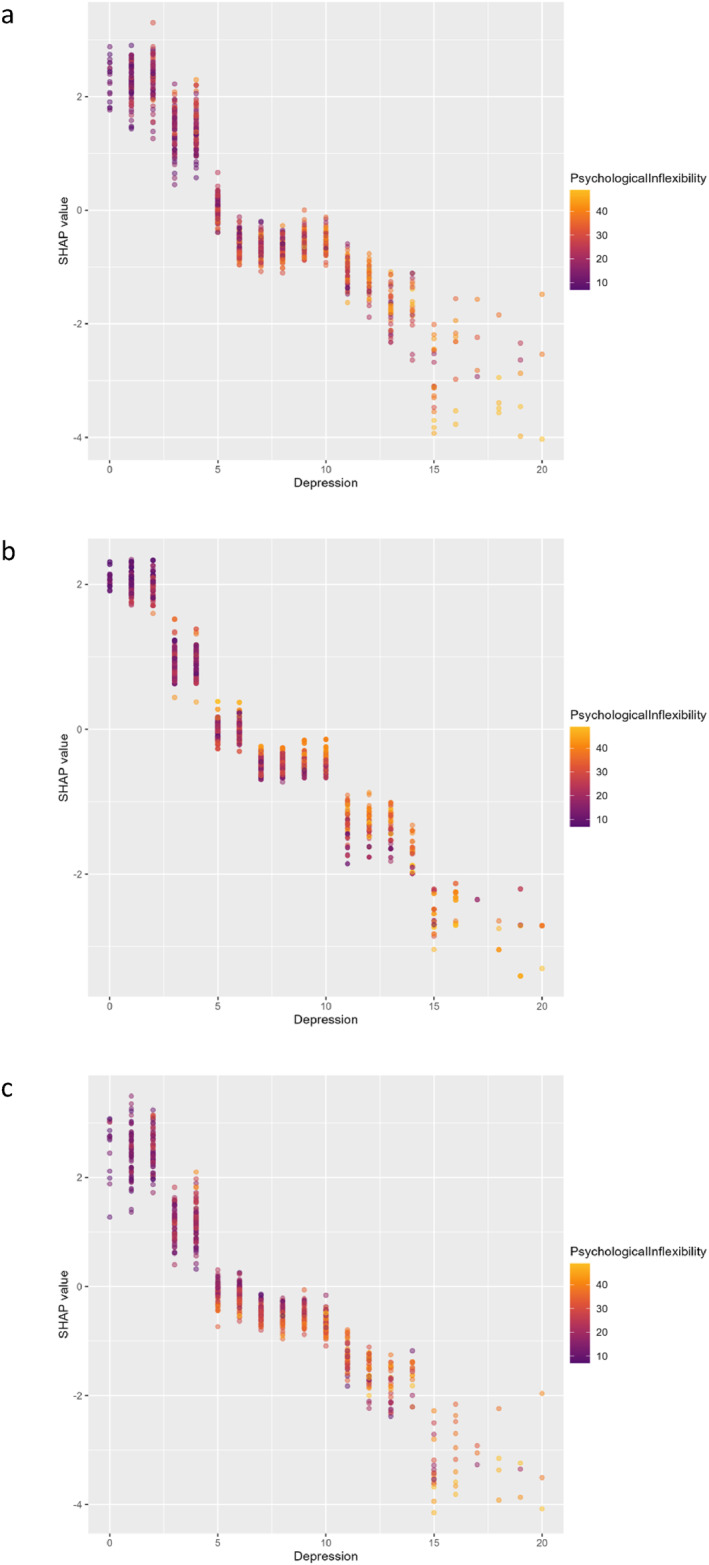
The nonlinear relationship between SHAP values of life satisfaction and depression, and the effect of psychological inflexibility on: (a) LightGBM, (b) XGBoost, and (c) Gradient Boosting.

Importantly, this negative effect was significantly amplified among individuals with higher psychological inflexibility: warmer colors (indicating higher inflexibility) show markedly more negative SHAP values at equivalent levels of depression. In contrast, individuals with lower psychological inflexibility (cooler colors) exhibit a more buffered decline, indicating that psychological inflexibility intensifies the detrimental association between depression and life satisfaction (Figure 5).

## 4 Discussion

### 4.1 Model Prediction

Various error metrics were employed to assess the relationship between the predicted and actual values while evaluating each model's estimation performance. The error metrics commonly used for regression model evaluation are the MAE and RMSE. Their values can range from 0 to infinity, where values closer to 0 signify more accurate predictions. The RMSE is particularly beneficial for larger datasets (Shen et al. 2023). Based on the MAE and RMSE values, XGBoost had the most prediction power. However, considering the approximate predictions of the three models, we evaluated all three models to reasonably predict life satisfaction.

Using different ML statistics algorithms to study life satisfaction has become widespread in recent years. Pan and Kutumisu pointed out that the multidimensional nature of life satisfaction and meaning in life, student competition, teacher support, exposure to bullying, and information and communication technology resources at home and school are important factors in predicting students' life satisfaction (Z. Pan and Cutumisu 2024). Shen et al. discussed the factors that predict life satisfaction in the elderly, regarding environmental, interpersonal, mood, and personality traits. The results have shown that subjective social status and positive and negative emotions are the most critical predictors of life satisfaction (Shen et al. 2023). Prati evaluated the life satisfaction of people over 50 in six dimensions––sociodemographic factors, personality, subjective life circumstances, activity, physical health, and childhood circumstances––finding physical health and subjective life circumstances to be the most predictive factors (Prati 2022).

### 4.2 Feature Importance

Based on all 3 ML algorithms, depression was the factor that most predicted life satisfaction, with high depression scores being negative predictors of life satisfaction (Figures 2 and 3). The relationship between depression and life satisfaction has been demonstrated many times in the literature, with numerous studies presenting a well‐established negative influence of depression on life satisfaction across diverse groups (Hofmann et al. 2017; Hoseini‐Esfidarjani et al. 2022; Romaniuk and Oniszczenko 2023). A regression analysis conducted among older individuals with cerebrovascular disease identified depression as the most crucial factor affecting life satisfaction as supported by our study (Chan et al. 2021). Furthermore, our SHAP interaction plots indicate that the effect of depression on life satisfaction is nonlinear (Figure 5). These plots demonstrate that SHAP values consistently trend downward as depression levels increase, showing that the model predicts a decrease in life satisfaction with rising depression. This decline is not linear; instead, it appears as a gradual acceleration of negativity. Notably, the sharp drop in SHAP values as depression levels shift from moderate to high suggests that the impact of depression on life satisfaction intensifies as levels increase.

The second most predictive factor for life satisfaction in all three models was PI, showing its importance for life satisfaction, being a transdiagnostic explanation for psychopathology that applies across different diagnoses. When an individual excessively focuses on negative internal experiences and structures their life around avoiding them, it leads to a restricted range of behaviors and the experience of PI. This cycle distances the person from important values that provide meaning in life, ultimately resulting in a life that lacks fulfillment and purpose. This conclusion is supported by some other studies concerning the negative impact of PI on life satisfaction (Alcaraz‐Ibáñez et al. 2017; Valdivia‐Salas et al. 2022). Beyond its direct effect, psychological inflexibility may also influence life satisfaction indirectly through its impact on depression. The relationship between PI and depression has been extensively documented in several studies (Yao et al. 2023). According to the PI perspective on psychopathology, feelings of depression may be a natural response. However, behaviors focused on avoidance in response to negative internal experiences can impede individuals from achieving their desired life and trap them in a destructive cycle, ultimately leading to depressive disorders (Kato 2016). The machine learning models revealed this moderating mechanism with particular clarity. SHAP interaction plots of all three models (XGBoost, LightGBM, and GBM) consistently reveal the following key pattern: Psychological inflexibility significantly moderates the non‐linear relationship between depression and life satisfaction. In other words, the negative impact of depression on life satisfaction becomes stronger or weaker depending on the level of psychological inflexibility. Particularly at high levels of psychological inflexibility, the negative effect of depression on life satisfaction is substantially amplified. We found a similar study in the literature that highlighted the moderating effect of psychological inflexibility. This study indicated that psychological inflexibility plays a moderating role in the relationship between depression and quality of life in breast cancer patients. Notably, this effect was observed at low or moderate levels of psychological inflexibility (Novakov 2021). In contrast, our study's SHAP interaction plots suggest that high psychological inflexibility intensifies the impact of depression on life satisfaction across nearly all depression scores. New studies examining these understudied relationships are needed. Taken together, these findings underscore the clinical importance of interventions targeting psychological inflexibility, such as ACT. Such approaches may not only directly enhance life satisfaction but also buffer against the detrimental impact of depressive symptoms on overall well‐being.

Previous research has shown that being married serves as a protective factor against different psychiatric disorders (Alzahrani et al. 2022; Hynek et al. 2022) and positively affects life satisfaction (Chan et al. 2021; Velten et al. 2014). In a study conducted in India on older adults and in China on those aged 45 and above, marriage significantly predicted life satisfaction positively (Nagargoje et al. 2022; L. Pan et al. 2022). In addition, Wortman & Lucas, suggested that divorced individuals experience a significant decline in life satisfaction compared to when they were married (Wortman and Lucas 2016). Our study supports these findings by highlighting the positive association between marital status and life satisfaction, with all 3 ML models showing marital status as the third important factor predicting life satisfaction. These results highlight the interpersonal aspect of life satisfaction, being married being a positive predictor. However, evaluating the marital situation by ignoring marital satisfaction may represent an incomplete assessment.

Based on the results of this study, procrastination is among the five most important factors predicting life satisfaction in all 3 ML models, although there have been conflicting results regarding the relationship between procrastination and life satisfaction. Procrastination can manifest in different ways––classed as functional or dysfunctional––complicating its relationship with life satisfaction. Several studies that have examined procrastination in a dysfunctional context have demonstrated a negative correlation between procrastination and life satisfaction, which is consistent with the findings of our study (Beutel et al. 2016; Yang 2021). Another study has suggested that the relationship between procrastination and life satisfaction is mediated by psychological stress (Maria‐Ioanna and Patra 2022). In this context, procrastination can be seen as an avoidance behavior in response to stress, resulting in a limited range of behaviors, as proposed by the PI model.

The social rhythm scale, which measures the regularity of daily circadian and social activities, emerged as an important contributor to life satisfaction in our study, being the fourth significant predictive factor in lightGBM and the fifth in XGBoost and GBM. This result is consistent with those in the previous literature covering different cultures and special groups. Two studies have reported morning chronotypes to have higher satisfaction in different cultures and ages (Jankowski 2012; Randler 2008). Regularity of social rhythm also positively predicts emotional well‐being (Cai et al. 2017). Velten et al.’s study, in which the scales and participants were similar to ours, also showed that irregular circadian and social rhythms negatively affected life satisfaction (Velten et al. 2014). In this context, the adverse effects of changing living conditions (e.g., technological developments, pandemics) on social and circadian rhythms should be taken seriously, and rhythm‐regulating tools should be given importance.

At the top of the list of factors affecting life satisfaction––the first five items––there was an apparent consistency between all three algorithms. Also, this alignment with previous studies reassures us of the robustness of our findings and instills confidence in our approach. Notably, the predictive powers of the rest of the list decreased, and there were differences between algorithms. Considering the position of anxiety versus depression in previous similar studies, and the relatively weak effects of exercise, alcohol use, smoking, and BMI factors, indicating lifestyle, it can be said that the data in the tables in our study are consistent with information in the literature (Mamani‐Benito et al. 2022; Ooi et al. 2022; Phulkerd et al. 2021; Velten et al. 2014).

### 4.3 Implications and Future Directions

Our study confirmed the complex nature of life satisfaction. Our findings highlighted that psychological factors—especially mood and psychological inflexibility—serve as more fundamental predictors of life satisfaction. Specifically, depression emerged as the primary issue to address to enhance life satisfaction. Achieving this requires overcoming prejudices and preventing stigmatization within the field of psychiatry, while ensuring access to effective treatment methods for everyone. The distinctive role of psychological inflexibility warrants particular attention, given its established position in the etiology of depression and its potential cascading effects on multiple determinants identified in this study. In this context, ACT, which seeks to transform a person's relationship with their negative internal experiences and empower them to live in accordance with their values, is becoming increasingly significant.

Among various lifestyle attitudes, the importance of social and circadian rhythms was particularly notable, while habits such as exercise, smoking, and alcohol consumption were less significant. This study emphasizes the importance of order and continuity in both individuals' private and social activities, rather than quantity. The fact that marriage ranked third among the most crucial factors, coupled with the importance of social regularity, underscores the role of social influences on life satisfaction.

### 4.4 Limitations

This study had some limitations. First, the non‐uniform gender distribution among the participants, with a greater willingness of women to participate in the online data collection, could potentially have impacted our findings. This underscores the need for a more balanced participant pool. Second, while lifestyle attitudes were relatively objectively assessed, the inability to comprehensively evaluate various lifestyle parameters as a composite score limited our insights into this aspect. Third, the cross‐sectional nature of our study limited the generalizability of the results. Fourth, social relations––potentially crucial factors for life satisfaction––were not evaluated using a specific scale in our study. Fourth, the reliability of the Turkish version of the Brief Social Rhythm Scale in our study was assessed using Cronbach's alpha, which achieved acceptable levels. However, confirmatory factor analysis revealed suboptimal model fit indices. This may limit the interpretability of findings related to social rhythms and suggests the need for scale refinement or adaptation for use in similar populations. Fifth, we evaluated the effect of marital status on life satisfaction, but marital satisfaction was not included. The relatively significant effect of marital status on life satisfaction also brings into question the effect of marital satisfaction. Finally, considering that depression, which has the most significant impact on life satisfaction, is influenced by various psychological and environmental factors, future studies should explore how the parameters examined in this study affect depression.

## 5 Conclusion

Two psychological factors—depression level and PI—are significant predictors of life satisfaction. Because it plays such a crucial role in enhancing life satisfaction, it is important to prioritize strategies that alleviate depression. Additionally, addressing PI is important because it not only serves as an independent therapeutic target, but it also influences the effect of depression. Among environmental factors, marriage and social rhythm have the most substantial impact on life satisfaction. These two factors also highlight the importance of interpersonal factors.

It was observed that factors that are impossible or relatively difficult to change, such as age, gender, education, and chronic disease, ranked relatively low on the list of predictors. ‐. In contrast, psychological and environmental factors that can be improved exhibited high predictive power. These results provide opportunities for better understanding life satisfaction and also influencing responsibility.

## Author Contributions

**Furkan Bahadir Alptekin:** conceptualization, writing – review and editing, supervision, writing – original draft, methodology, data curation, resources, project administration. **Ebrar Torlak:** conceptualization, supervision, investigation, data curation. **Özge Asik:** conceptualization, supervision, investigation, data curation. **Betul Karaaslan:** conceptualization, supervision, investigation, data curation. **Ebru Turgal:** conceptualization, supervision, investigation, methodology, formal analysis, data curation. **Huseyin Sehit Burhan:** conceptualization, supervision, methodology, project administration, validation. **Hasan Mervan Aytac:** conceptualization, supervision, funding acquisition, project administration, validation. **Oya Guclu:** conceptualization, supervision, project administration.

## Funding

The authors have nothing to report.

## Ethics Statement

Ethical committee of Başakşehir Çam and Sakura City Hospital Clinical Research Ethics Committee approved the study (2023‐400). Informed consent was obtained from participants. All procedures were in accordance with the ethical standards of the institutional and national research committee and with the 1964 Helsinki declaration and its later amendments or comparable ethical standards.

## Conflicts of Interest

The authors declare no conflicts of interest.

## Data Availability

Data is available upon reasonable request.
